# Near-complete genome sequence of human astrovirus recovered from a child with acute flaccid paralysis in Guinea, 2021

**DOI:** 10.1128/MRA.00214-23

**Published:** 2023-09-29

**Authors:** Ousmane Kèbè, Fatou Diène Thiaw, Ndack Ndiaye, Dadja Essoya Landoh, Gassim Cissé, Ousmane Faye, Martin Faye

**Affiliations:** 1 Inter-country WHO reference laboratory for poliomyelitis, virology department, Institut Pasteur de Dakar, Dakar, Senegal; 2 WHO country office, Conakry, Guinea; 3 Ministry of Health and Public Hygiene, Conakry, Guinea; Katholieke Universiteit Leuven, Leuven, Belgium

**Keywords:** human Astrovirus, acute flaccid paralysis (AFP), Guinea, West Africa

## Abstract

Astroviruses are common causes of gastroenteritis in humans and other animals. Herein, we reported a near-complete human astrovirus (HAstV) sequence detected in a child with acute flaccid paralysis. The sample was collected in Guinea in January 2021. Phylogenetic analyses indicated that this virus belonged to the HAstV-1 genotype.

## ANNOUNCEMENT

Belonging to the *Astroviridae* family, astroviruses are non-enveloped viruses responsible for acute gastroenteritis (AGE) worldwide and are associated with 2–8% of childhood diarrhea in Africa (1). Human astrovirus (HAstV) infections are mostly characterized by mild, watery diarrhea lasting 2 to 4 d and, in some cases, associated with abdominal pain, vomiting, headache, fever, and anorexia (2, 3). However, severe dehydration leading to hospitalization has also been described in some cases (4–7). In addition, HAstV is increasingly associated with fatal encephalitis and meningitis in humans (8), mainly in immunocompromised patients (9–12).

The viral genome is a single-stranded, positive-sense RNA of approximately 6,700 to 7,000 nucleotides in length, which is divided into three overlapping open reading frames (ORFs) (13–16). The ORF1a and ORF1b encode for non-structural proteins such as the protease and the polymerase, respectively. The ORF2 encodes the viral structural protein (4) and is commonly targeted for genotyping of astroviruses (17–20). Previous studies have reported the presence of HAstV MLB1 in Nigeria and Pakistan and HAV1 in Iraq in stool samples collected from non-polio acute flaccid paralysis children (17–20). In this study, we present the near-complete genome sequence of a HAstV-1 strain detected in a child with AFP in January 2021.

**Fig 1:**
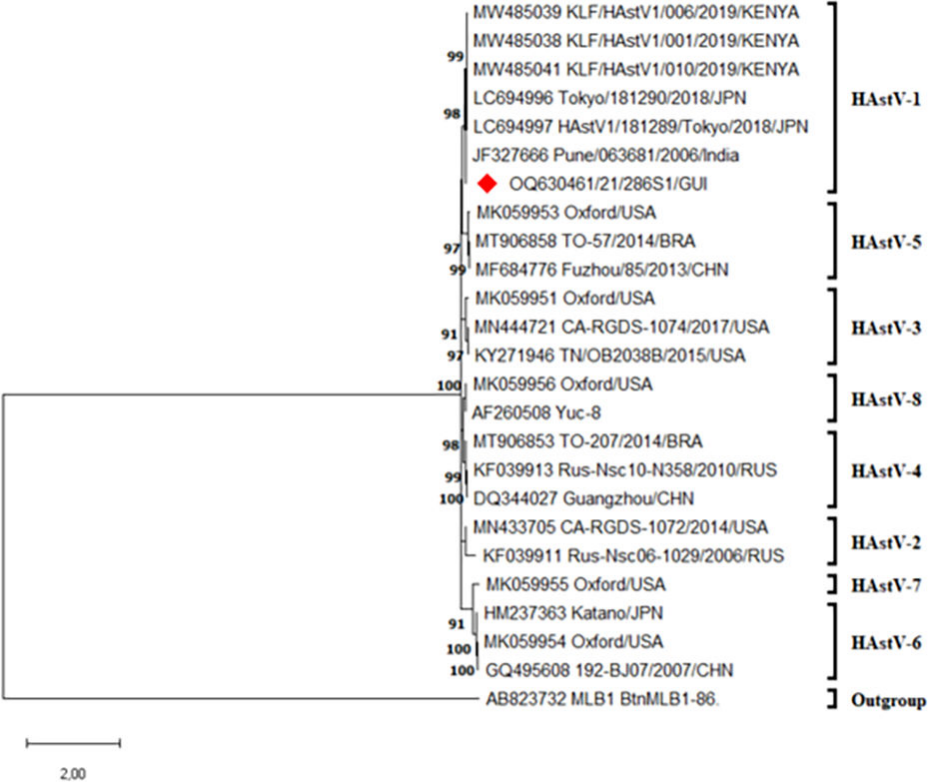
Maximum-likelihood phylogenetic tree of the HAstV-1 SEN_21_286 genome sequence based on the 6389-nt sequence corresponding to nt positions 147–6,533 of the most closely related HAstV strain from India (JF327666). The red triangle symbol indicates the newly characterized strain.

The HAstV-1 strain (SEN_21_286_S1) was detected in January 2021 in a child with AFP living in the Matoto district in the Conakry region of Guinea. The patient was a 1-year-old girl with an onset of symptoms on 04 January 2021, including mainly flaccid paralysis in the lower limbs. Two stool samples were collected on 13 January 2021 (SEN_21_286_S1) and 14 January 2021 (SEN_21_286_S2) in the framework of AFP surveillance activities in support of the global polio eradication initiative and sent to the inter-country WHO reference laboratory for poliomyelitis surveillance at Institut Pasteur de Dakar in Senegal. The samples tested negative for polioviruses and non-polio enteroviruses according to the WHO-recommended algorithm for poliovirus isolation (21). Only the sample SEN_21_286_S1 was selected and thereafter analyzed by metagenomic next-generation sequencing. After viral RNA extraction from 140 µL of clarified stool suspension using the QIAamp viral RNA kit (QIAGEN, Hilden, Germany), the first-stranded cDNA was synthesized from the depleted ribosomal RNA and DNAse-treated RNA using the SuperScript IV Reverse Transcriptase first-strand synthesis supermix kit (Invitrogen, Thermo Fisher, USA), and the double-stranded cDNA was produced using the MyTaqRed (Bioline, USA). The libraries were prepared using the Nextera XT library preparation kit (Illumina, USA) according to the recommendations of the fabricant. The generated library, with an average size of 350 base pairs, was tagged using the Nextera XT indexes kit (Illumina, USA) and diluted to a final concentration of 2 nM. The 2 nM library was sequenced with paired-end reads using the Illumina MiSeq reagent kit v2 (300 cycles) on an Illumina MiSeq instrument. A total of 96,532 reads were obtained for the sample SEN_21_286_S1**,** and 5.93% of the reads (*n* = 5,733) were 100% aligned to the human astrovirus using bowtie2 (accession number JF327666). The quality control was done after aligning the reads using fastQC, and the consensus genome was generated using the *de novo* assembly method implemented on the fully open-source EDGE Bioinformatics software (22). A nearly complete genome sequence of 6,389 bp was obtained. After BLASTn analyses, the genome was shown to be most closely related (97% nt similarity) to an Indian human astrovirus strain Pune/063681/India (accession number JF327666), isolated from a patient suffering from AGE in 2006. A Maximum-Likelihood phylogenetic tree, based on the complete genome sequence of the newly characterized isolate and previous sequences available from GenBank, was inferred using the MEGA 11 program (23) for 1,000 replications. The tree was constructed using the general time-reversible (GTR) substitution model with a gamma distribution (+G). The phylogenetic tree was unrooted, and nodes were supported by the bootstrap values; only values >70 were shown on the tree (Fig. 1).

The newly detected HAstV-1 sequence in Guinea clustered closely with the abovementioned Indian strain in a cluster together with other genotype 1 strains, represents, to date, the first near-complete sequence from West Africa.

## Data Availability

The data that support the findings of this study are available from the corresponding author upon reasonable request. The HAstV-1 SEN_21_286 genome sequence has been deposited in GenBank with the accession number OQ630461. The raw data were deposited to Sequence Read Archive (SRA) (https://dataview.ncbi.nlm.nih.gov/object/PRJNA991560?archive=sra) with the accesssion number SRR25194097.
